# Editorial: Myokines, Adipokines, Cytokines in Muscle Pathophysiology, Volume II

**DOI:** 10.3389/fphys.2022.907956

**Published:** 2022-05-30

**Authors:** Valentina Di Felice, Dario Coletti, Marilia Seelaender

**Affiliations:** ^1^ Department of Biomedicine, Neurosciences and Advanced Diagnostics, University of Palermo, Palermo, Italy; ^2^ Sorbonne Université, Institut de Biologie Paris-Seine, (IBPS), CNRS UMR 8256, Inserm ERL U1164, Biological Adaptation and Ageing, Paris, France; ^3^ Department of Anatomical, Histological, Forensic Sciences and Orthopedics, Sapienza University of Rome, Rome, Italy; ^4^ Department of Anatomical, Histological, Forensic Sciences and Orthopedics, Interuniversity Institute of Myology, Rome, Italy; ^5^ Department of Surgery, Cancer Metabolism Research Group, LIM 26-HC, Faculty of Medicine, University of São Paulo, São Paulo, Brazil

**Keywords:** skeletal muscle tissue, myokines, adipokines, cytokines, organ cross-talk

## Introduction

The maintenance of tissue homeostasis, the coordination of organ functions and the response to pathological insults rely on inter-organ crosstalk. Myokines, adipokines, and cytokines are major peptide mediators of such complex conversation among compartments (de Matos-Neto et al., 2015; Neto et al., 2018). In a previous Research Topic dedicated to this subject we edited a collection of papers discussing the role of these factors in promoting muscle stem cell function and muscle regeneration; as they mediate the effects of exercise upon the muscle and other organs; and their role in disease, when altered levels of these important mediators are described in pathological conditions (Di Felice et al., 2020). With this second Research Topic we expand the discussion, adding to the Research Topic of muscle pathophysiology and inter-organ crosstalk, with a new Research Topic of original papers.

### New Insights on Myokines, Adipokines, Cytokines and Non-Coding RNAs modulation of Muscle Pathophysiology

Myokines, adipokines and cytokines are circulating factors regulating muscle homeostasis in paracrine and/or endocrine fashion (Baccam et al., 2019). Both myokines and non-coding RNAs are carried by extracellular vesicles (Trovato et al., 2019), with important implications for health and disease (Sibley and Wood, 2011). The group of Sampaolesi proposes the novel and exciting possibility of manipulating miRNA in order to treat muscle tumors. The authors show that miR181a/miR212 improve myogenic commitment, thus reducing cell proliferation in rhabdomyosarcomas, the most common soft tissue sarcomas (Pozzo et al.). Isesele and Muzarak shed light on the recent findings that the proliferation and differentiation of normal muscle myoblasts, i.e. the satellite cells, are also regulated by non-hormonal factors, such as Omega-3 polyunsaturated fatty acids (PUFAs), in the elegant review presented. Interestingly, miRNAs and PUFAs can actually interact within the same pathways, with important implications for cancer prevention (Carotenuto et al., 2016a) and for muscle regeneration and homeostasis (Carotenuto et al., 2016b; 2016c). These interesting aspects argue for a potential impact of diet in the mediation of humoral crosstalk. Adding to the discussion on the complexity of the regulation and orchestration of signals, Cheng points out that miRNAs and cytokines act within an interactive network, with important consequences on muscle regeneration, a process highly affected by the presence of inflammation (Cheng et al., 2020), a common feature in chronic diseases.

In an original approach Renzini et al. point out that histone deacetylases (HDACs) are also relevant modulators participating in the crosstalk between the skeletal muscle and other organs (Renzini et al.). Interestingly, besides being activated by adipo-myokines in muscle tissue, HDACs, in turn, affect the synthesis of myokines by the skeletal muscle, thereby creating a signaling loop. As a consequence, it is not surprising that HDAC4, for instance, plays a major role in muscle homeostasis and repair under pathological conditions (Renzini et al., 2022).

Another interesting cytokine-induced reversible modification that occurs in the musculature is the O-GlcNAcylation of proteins. This modification has broad effects on muscle tissue, inducing structural (Lambert et al., 2020) and metabolic (Lambert et al., 2018) modification of the myofibers. The group of Gerrard reports that O-GlcNAc transferase action on global metabolism is mediated through IL-15, leading to a lean phenotype in models (Zumbaugh et al.). Arginine methylation, mediated by protein arginine methyltransferases (PRMTs), is yet another post-translational modification of both histone and non-histone proteins, which is critical for muscle physiology, as pointed out by the group of Lee (So et al.). PRMTs play a major role in skeletal muscle during inflammation, which, affects skeletal muscle homeostasis and health (Coletti et al., 2013; Bouché et al., 2014; Gonçalves et al., 2021).

Exercise remains a major stimulus for myokine release (Barone et al., 2017), which is often mediated through the shedding of extracellular vesicles (Trovato et al., 2019). An increase in circulating small extracellular vesiscles enriched in Hsp60, a new myokine (Gammazza et al., 2018), has been demonstrated in trained animals (Barone et al., 2016). Exercise has many systemic beneficial effects (Coletti et al., 2016; D’amico et al., 2021; de Lima et al., 2020; Macaluso et al., 2013; Pigna et al., 2016), among which, the establishment of an anti-inflammatory milieu and the increased release of myokines (Scheffer and Latini, 2020). The latter are precious tools for the treatment of several muscle and non-muscle diseases (de Castro et al., 2021). In the era of gene therapy, it is also possible to conceive gene delivery-based approaches aimed at increasing the sensitivity of the skeletal muscle to circulating humoral factors (Toschi et al., 2011) known to promote muscle regeneration and homeostasis (Musarò et al., 2007; Coletti et al., 2013).

## Conclusion

The complex crosstalk involving myokines, cytokines, metabolites, non-coding RNAs and enzymes (both in soluble and vesicle-bound forms) is being progressively unveiled, providing the basis for manipulation of the levels these mediators. Exercise, nutritional supplementation or pharmacological treatment all of which induce changes in the signaling orchestra, appear to have major potential for the treatment of many muscle and non-muscle diseases. Organ crosstalk deserves unreserved attention in agreement with the modern, holistic view of the patient and the diffusion of multimodal approaches for personalized medicine. A better understanding of the role of humoral factors and their relationship with other mediators taking part in one such crosstalk grants improved knowledge of muscle pathophysiology and, as a consequence, pave the way to new therapeutical interventions.
